# Prevention of Postsurgical Abdominal Adhesion Using Electrospun TPU Nanofibers in Rat Model

**DOI:** 10.1155/2021/9977142

**Published:** 2021-12-28

**Authors:** Ahmad Gholami, Homeira Emad Abdoluosefi, Elham Riazimontazer, Negar Azarpira, Mohamadali Behnam, Farzin Emami, Navid Omidifar

**Affiliations:** ^1^Biotechnology Research Center, Shiraz University of Medical Sciences, Shiraz, Iran; ^2^Pharmaceutical Sciences Research Center, Shiraz University of Medical Science, Shiraz, Iran; ^3^Department of Nuclear Physics, Faculty of Physics, University of Kashan, Kashan, Iran; ^4^Department of Medicinal Chemistry, School of Pharmacy, Shiraz University of Medical Sciences, Shiraz, Iran; ^5^Organ Transplant Research Center, Shiraz University of Medical Sciences, Shiraz, Iran; ^6^Nano Opto-Electronic Research Center, Electrical and Electronics Engineering Department, Shiraz University of Technology, Shiraz, Iran

## Abstract

Intra-abdominal adhesions following surgery are a challenging problem in surgical practice. This study fabricated different thermoplastic polyurethane (TPU) nanofibers with different average diameters using the electrospinning method. The conditions were evaluated by scanning electron microscopy (SEM), atomic force microscope (AFM), and Fourier transform infrared spectrometer (FTIR) analysis. A static tensile test was applied using a strength testing device to assess the mechanical properties of the electrospun scaffolds. By changing the effective electrospinning parameters, the best quality of nanofibers could be achieved with the lowest bead numbers. The electrospun nanofibers were evaluated *in vivo* using a rat cecal abrasion model. The macroscopic evaluation and the microscopic study, including the degree of adhesion and inflammation, were investigated after three and five weeks. The resultant electrospun TPU nanofibers had diameters ranging from about 200 to 1000 nm. The diameters and morphology of the nanofibers were significantly affected by the concentration of polymer. Uniform TPU nanofibers without beads could be prepared by electrospinning through reasonable control of the process concentration. These nanofibers' biodegradability and antibacterial properties were investigated by weight loss measurement and microdilution methods, respectively. The purpose of this study was to provide electrospun nanofibers having biodegradability and antibacterial properties that prevent any adhesions or inflammation after pelvic and abdominal surgeries. The *in vivo* experiments revealed that electrospun TPU nanofibers reduced the degree of abdominal adhesions. The histopathological study confirmed only a small extent of inflammatory cell infiltration in the 8% and 10% TPU. Conclusively, nanofibers containing 8% TPU significantly decreased the incidence and severity of postsurgical adhesions, and it is expected to be used in clinical applications in the future.

## 1. Introduction

Surgical intra-abdominal adhesions are a common cause of postoperative complications, which is found in most cases of abdominal and gynecological surgeries [1]. Abdominal and pelvic adhesions typically form pathological bonds between defect areas such as damaged peritoneal surfaces and other tissues that come in contact with the visceral peritoneum in the uterus, ovaries, uterine tubes, and intestines [2]. These adhesions remain one of the most challenging problems in surgical procedures, and despite many efforts in surgical techniques, there is still no reliable strategy to manage them. It is the main unpleasant consequence of abdominal procedures. The effects of adhesion formation are long-term complications such as chronic abdominal and pelvic pain, bowel obstruction, female infertility, and difficulty with future operations [3]. Accordingly, surgical adhesions will have a profound economic effect. It has been estimated that many patients are admitted to hospitals annually due to adhesion removal surgery and bowel obstruction surgery, accounting for inpatient care and medical expenditures and contributing to many deaths annually [3]. The best approach to reduce the severity of complications, limit morbidity, and decrease the economic cost is to prevent the formation of postoperative adhesions.

Some common prior strategies are applying antiadhesion materials and fibrinolytic agents and improving surgical procedures [4]. A method that is widely used is placing physical barriers between the injured site and adjacent tissues. They are applied to cover the injured peritoneal tissues during mesothelial regeneration and prevent the adherence of adjacent structures and adhesion formation [5]. Some of the disadvantages of physical barriers are the inability to cover all visceral and peritoneal surfaces, increased risk of intra-abdominal abscesses, prolonged operative time, and higher cost [5, 6]. Animal membranes, gold foils, mineral oil, silk, rubber, and Teflon sheets are the common barrier materials with only limited success.

Nanofibers are a large category of nanosized fibers with various physicochemical properties made from different polymers, which were successfully used in many biomedical sciences such as tissue engineering, wound coatings, physical barriers, and drug delivery [7, 8]. Thermoplastic polyurethane (TPU) elastomers are the most common nanofibers composed of a linear class of copolymers and characterized by the presence of carbamate groups [9].

Nanofiber formation through electrospinning is a versatile and straightforward technique using biopolymers and nanocomposites that have gained much attraction to provide a proper environment [10, 11]. Further, the nanodimension of the fiber naturally gives it a high surface area to volume ratio, leading to the facility of control over the diameter, structure, and morphology of the electrospun fibers [12, 13]. Electrospinning applied a high voltage on a contained polymer solution to generate a repulsive force on the charged solution that accelerated through a spinneret toward the collector [10]. Then, the target-forming nanofibers are deposited on the collector. Due to achieving fine or even ultrafine nanofibers with the optimal mechanical properties, several parameters and processing variables should be controlled, including solution properties (viscosity, concentration, molecular weight, conductivity), processing conditions (applied voltage, distance from needle to collector), and ambient conditions (temperature, humidity) [14–16]. In electrospinning, TPU nanofibers showed high resistance to mechanical degradation over time when exposed to high-stress conditions and have acceptable biocompatibility with tissues [17]. Degradation of the nanofibers is one of the most critical issues and needs to be investigated. Biodegradable nanofibers could metabolize into biocompatible, nontoxic products in the human body; therefore, a second surgery for implant removal is unnecessary [18, 19]. It is worth noting that TPU nanofibers have significant antimicrobial activity [20]. Bacterial infections, especially surface bacteria such as *E. coli* and *Staphylococcus aureus*, play an essential role in the progression and consequences of surgery, especially in this age when engineered bacteria are almost everywhere [21–23]. Antibacterial properties are among the essential properties a substance must have to perform the best wound healing, especially after surgery [24]. Nanofiber scaffolds from polyurethane (PU) can effectively antiadhesion as these substances possess their specific therapeutic properties, including good mechanical properties, good barrier properties, biodegradability, biocompatibility, antibacterial properties, and oxygen permeability [25, 26].

Although new materials have been selected to satisfy the standards for adhesion prevention, none of them have met all the desirable features. So, there is still a need for an advanced and inexpensive membrane to be effectively used in surgeries for a wide range of patients to reduce postoperative adhesions. In this work, some nanostructured barriers made from TPU were fabricated by the electrospinning technique in different polymer solution concentrations. The nanofibers' morphology, size, and structure in different concentrations were analyzed by various characterization techniques. The mechanical properties, biodegradability, and antibacterial feature of the samples were also studied by strength testing device, weight loss measurement, and microdilution method, respectively [18]. Then, the *in vivo* antiadhesion efficacy of nanofibers compared to routine surgical mesh was evaluated in a rat cecal abrasion model to demonstrate the possible application for the antiadhesion property.

## 2. Materials and Methods

### 2.1. Materials

Commercial medical-grade thermoplastic polyurethane (MW: 120 kDa, Bayer AG, Germany) was used as a raw material. Tetrahydrofuran (THF, Samchun Pure Chemical Co., South Korea) and N,N-dimethylformamide (DMF, Samchun Pure Chemical Co., South Korea) were purchased solvents to prepare the electrospun nanofibers. Commercial soft polypropylene nonabsorbable synthetic surgical mesh (PROLENE® Soft Polypropylene Mesh, Ethicon, Cincinnati, OH, United States) and phosphate-buffered saline (PBS) were purchased from the Sigma-Aldrich Company, St. Louis, US.

### 2.2. Preparation of the Electrospun TPU Nanofiber Scaffolds

Structures of electrospun TPU nanofibers were fabricated through the uniaxial electrospinning technique. The electrospinning device (Asian Nanostructures Technology Company, Iran) consists of a voltage regulating system up to 30 kV, which could adjust the flow rate of the solution through the uniaxial needle. The rotation speed of the collector (in rpm) and the possibility to set the appropriate distance between collectors and needle were under control. Different concentrations of TPU solutions (6%, 8%, 10%, and 12% *W*/*V*) were prepared in such a way that 0.6, 0.8, 1, and 1.2 g of the TPU was dissolved in 4 mL of DMF and 6 mL of THF and stirred to obtain a homogenous solution at 25°C. Solutions of TPU were embedded in a 10 mL syringe with an internal radius of 23 gauges and were fed through the needle to create stable fibers. Nanofibers were electrospun at a rotation speed of 500 rpm, 19 kV voltage, and 1.5 mL/h flow rate, whereas the distance between syringe tip and collector was 140 mm at room temperature (Table 1). After optimizing all essential parameters, the electrospinning process was started, and polymeric nanofibers were collected on a sheet of aluminum foil wrapped around a rotating drum.

### 2.3. Characterization of Electrospun TPU Nanofibers

The morphology of the electrospun TPU nanofibers was observed using scanning electron microscopy (SEM, Philips XL-30, USA) at an accelerating voltage of 15 kV. A total of 100 fibers were counted for each sample, and diameters and distributions of the electrospun nanofibers were measured by the SEM images using the Digimazer image analysis software version 4.3.5.

Atomic force microscope (AFM, Nanosurf AG NaioAFM, Switzerland) measurements were performed to determine the morphology and topographic properties of TPU nanofibers.

Fourier Transform Infrared spectrometer (FTIR, Vertex70, Bruker) was used to determine the chemical properties of nanofibers.

To assess the mechanical properties of the electrospun scaffolds, a static tensile test was applied using a strength testing device (Zwick/Roell Z250, Germany). All samples were cut into strips with the gauge 6 mm width dimension by 0.03 mm length. 0.2 N measuring cell was employed under tensile loading at 37°C in a temperature chamber and 70% relative humidity with an elongation speed of 10 mm/min.

### 2.4. Animal Handling

The antiadhesion characteristics of the TPU nanofibers were investigated using a rat cecal abrasion model. The procedures and handling of the animals were performed according to the guidelines approved by the Ethics Committee (IR.SUMS.REC.1398.072) of Shiraz University of Medical Sciences, Shiraz, Iran. The *in vivo* study to assess the antiadhesion efficacy of electrospun TPU nanofibers in the abdominal cavity was carried out using 96 healthy female Sprague Dawley rats (divided into 12 groups (*n* = 8)) aged at 8-10 weeks with an average body weight of 200–250 g. The rats were purchased from the Laboratory Animal Centre of Shiraz University of Medical Sciences. The rats' observation was done upon coming to the laboratory for a week to adapt to the new place. Throughout the experiment, rats were housed in standard plastic cages at 23-25°C and 40–60% relative humidity with a controlled 12-hour light/dark cycle for at least two weeks before the experiment. Standard pellet diet and water were freely available for the animals throughout the investigation, and there was no difference between the experimental groups.

### 2.5. Animal Antiadhesion Studies

An aseptic condition was provided for the surgical procedure. Anesthesia was performed intramuscularly in all groups using a combination of two drugs, ketamine (80 mg/kg) and xylazine (10 mg/kg). After anesthesia, the rats were placed on the surgical table in the supine position, and the surgical field was prepared with 1% of antiseptic povidone-iodine [27]. The hair was shaved entirely on the midline of the abdomen under sterile surgical conditions. After entering the right wall of the abdominal area by razor blade no. 24.3, shallow slices were created longitudinally and transversely on the left side of the abdominal wall. This was done by surgical scissors from the peritoneal surface and each piece with 4 × 4 cm dimensions. All 4 groups of nanofibers were immersed in phosphate-buffered saline (PBS) for a few minutes and then implanted in the abdomen, and then, the abdomen was closed with 4–0 silk sutures.

**Figure 1:**
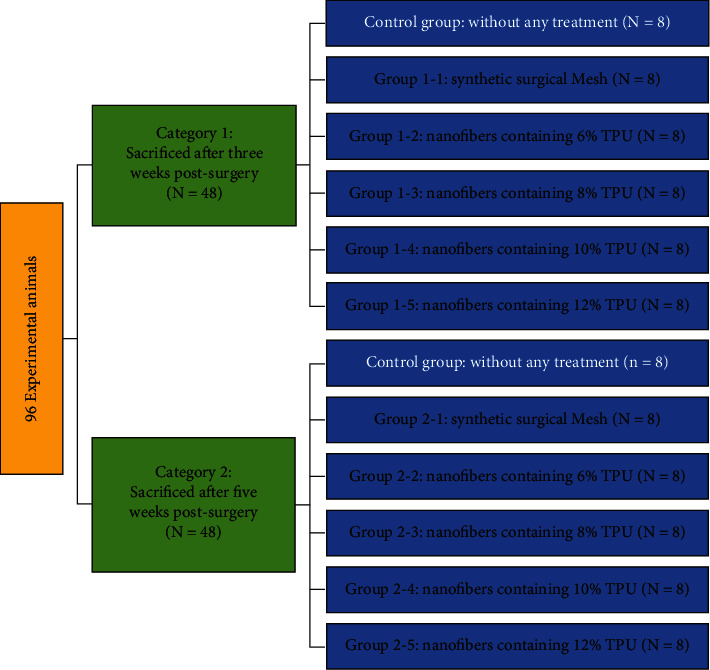
Schematic diagram of the classification of experimental rats in 10 groups. The classification of 80 experimental animals into 2 experimental categories contains 5 experimental groups with 8 rats in each group. We compared cytokine levels 3 weeks and 5 weeks after experimental abdominal surgery to a control group.

For each group of rats, one type of electrospun TPU nanofibers was implanted. The rats were divided into two categories; each category included five groups (*n* = 8, Figure 1). In category 1, in which animals were sacrificed after three weeks postsurgery, group 1 was the standard group with a commercial soft polypropylene nonabsorbable synthetic surgical mesh (PMS Stripsck 20 cm × 70 cm) for comparison as a standard, with group 2 nanofibers containing 6% TPU, group 3 nanofibers containing 8% TPU, group 4 nanofibers containing 10% TPU, and group 5 nanofibers containing 12% TPU. This grouping was also repeated in category 2. Nanofibers' macroscopic and microscopic characteristics were assessed three weeks postsurgery in category 1 and five weeks in category 2. In both categories, a group of animals that did not receive any treatment after surgery was considered the control group.

### 2.6. Macroscopic Evaluation

The scaffolds of TPU nanofibers in different concentrations were implanted into the body subcutaneously compared to a control group. At the end of each course (three and five weeks), the rats were sacrificed, and the adhesion to surrounding tissue and its shape was evaluated macroscopically. The formation of adhesion was assessed according to a semi-quantitative scoring system as follows [28]: no adhesion = score 0; one thin filmy adhesion easily separable with blunt dissection: score 1; definitely localized adhesions with free dissection plane: score 2; dense multiple visceral adhesions: score 3; dense adhesions extending abdominal wall: score 4.

Abdominal adhesions were assessed by two independent persons blinded to experiments. Then, the grade scores were measured using the following equation:

(1) $$\begin{array}{c} \text{Grade score}=n*\frac{G}{N}, \end{array}$$

wherein *n* is the number of rats with the same grade score in each group, *G* is the macroscopic score, and *N* is the total number of rats in each group. Finally, the summary of all scores obtained in each group is reported in Table 2.

### 2.7. Histological Analysis

After an animal sacrifice and macroscopic evaluations, the surgery site was excised and collected for histological evaluation. The tissues were fixed in 4% formalin for 24 hours and embedded in formalin. Samples were cut into 5 *μ*m sagittal slices and stained with H&E. The stained sections were examined by an independent and blinded investigator with a CKX3 Olympus microscope (Japan), and images were captured [29]. Histological findings were scored in two subcategories consisting of cell and tissue morphology of the capsule and the surrounding tissue components of the capsule, according to Bölgen et al. [30]. The tissue response was scored and expressed as means ± SD. The scoring criteria have been described in Table 1.

### 2.8. Degradation Analysis

The synthesized electrospun TPU nanofibers in different concentrations and synthetic surgical mesh as a standard in dimensions of 1 × 20 × 80 mm pieces were weighed (*W*_1_) and immersed in a PBS buffer (pH: 7.4) containing penicillin-streptomycin (100 U/mL) and incubated for 1, 2, 4, 8, 16, 32, and 64 days at 37°C. All samples were then washed with double distilled water and dried in a vacuum oven. At the end of each period, the weight of the samples was calculated in milligram (*W*_2_), and the percentage of weight loss in the respected time was determined using the following formula:

(2) $$\begin{array}{c} \text{The percentage of weight loss }\left(\%\right)=\frac{W_{1}-W_{2}}{W_{1}}*100. \end{array}$$

For each degradation time, this experiment was done in triplicate [18].

### 2.9. *In Vitro* Assay of Antibacterial Activity

*Staphylococcus aureus* and *Escherichia coli*, as two pathogenic microorganisms, were selected for the antibacterial assay of TPU according to Heiran et al. [31]. The organisms were suspended in freshly prepared Mueller-Hinton's broth (MHB) at a standard concentration of 0.5 McFarland and diluted with a 1 : 20 proportion by MHB. An aqueous solution of different concentrations of electrospun TPU nanofibers was prepared so that the range of concentration would contain 0.05 mg/mL to 10 mg/mL of compounds. A 96-well microplate consisting of 45 *μ*L culture media, 45 *μ*L of the sample (at a descending concentration of compounds from 10 mg/mL to 0.05 mg/mL), and 10 *μ*L of inoculated bacteria was applied for each microorganism. The first and last rows of the microplate were left empty to achieve a better optical contrast after plate reading. The prepared microplates were incubated for 24 hours at 37°C; then, the optical density was measured at 600 nm by a microplate reader (BioTek, PowerWave XS2). This procedure was repeated three times. A blank 96-well microplate consisting of 45 *μ*L of culture media and 45 *μ*L of the sample (as explained) was prepared. In the end, 10 *μ*L of culture media was added to each well. The turbidity of each well in a sample microplate (a microplate with the concerned microorganism) was compared to an equivalent well in a blank microplate. Microorganism viability was calculated as follows:

(3) $$\begin{array}{c} \%\text{microorganism viability}=\frac{{\text{OD}}_{\left(\text{bacteria}+\text{sample}\right)}-{\text{OD}}_{\left(\text{Sample}\right)}}{{\text{OD}}_{\left(\text{bacteria}\right)}-{\text{OD}}_{\left(\text{RPMI}\right)}}\times 100. \end{array}$$

All antimicrobial studies were evaluated using IBM SPSS software.

### 2.10. Statistical Analysis

The data obtained from macroscopic and microscopic evaluations and histological analysis were analyzed by Kruskal–Wallis one-way analysis of variance and Mann–Whitney *U*-tests using statistical software SPSS IBM.; *p* value <0.05 was considered significant. The number of animals in each group was 8, and the data were expressed as means ± SD.

To determine the differences between the means of degradation and antimicrobial assay results, the one-way ANOVA procedure and the post hoc Tukey test were performed. *p* value ≤0.05 was considered to be statistically significant. The experiment was repeated three times.

## 3. Results and Discussion

### 3.1. Morphology of Electrospun TPU Nanofibers

Several processing parameters are involved in the fabrication of nanofibers by the electrospinning method. These parameters, including applied voltage, the flow rate of the solution, and needle tip to collector distance, should be optimized to obtain the desired nanofibers. In this study, the optimum parameters for nanofiber preparation are as follows in Table 3.

**Figure 2:**
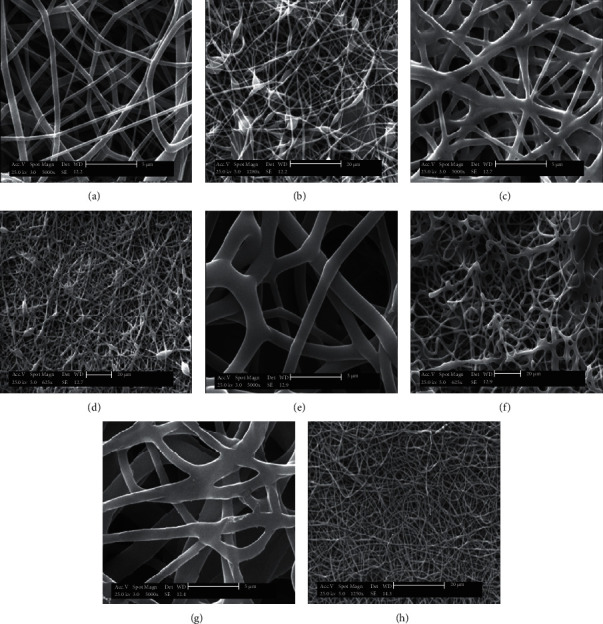
SEM images of electrospun TPU nanofibers at different concentrations: (a, b) 6% *W*/*V*, (c, d) 8% *W*/*V*, (e, f) 10% *W*/*V*, and (g, h) 12% *W*/*V*.

**Figure 3:**
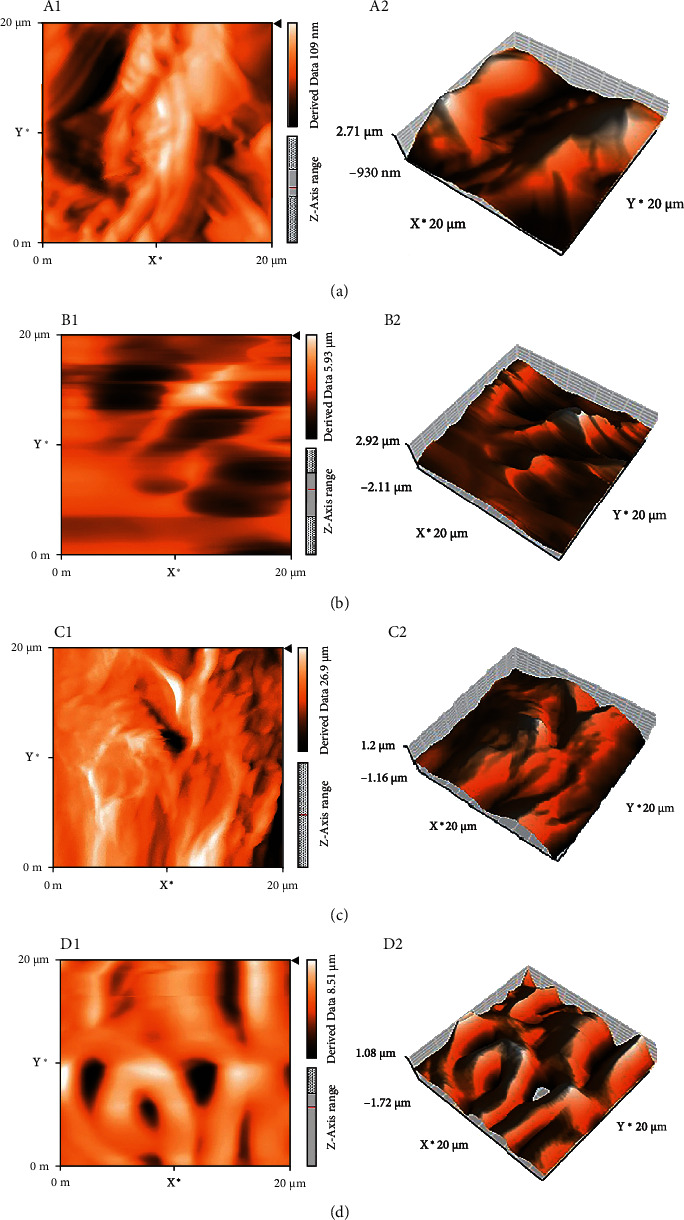
AFM images of electrospun TPU nanofibers at different concentrations: (A1, A2) 6% *W*/*V*, (B1, B2) 8% *W*/*V*, (C1, C2) 10% *W*/*V*, and (D1, D2) 12% *W*/*V*.

Then, the effects of changing the concentration of polymer solutions (ranging from 6% to 12% *W*/*V*) on the chemomechanical properties and biological activity of fabricated nanofibers were investigated. The SEM images and morphologies of the resulting nanofibers in two different magnifications are shown in Figure 2. Topography, surface properties, and the average height of TPU nanofibers were studied by AFM microscopy. As shown in Figure 3, quantitative roughness measurements obtained from AFM revealed that all fabricated electrospun nanofibers (TPU 6%, TPU 8%, TPU 10%, and TPU 12% *W*/*V*) had smooth surfaces.

The cross-sectional morphology of nanofibers also exhibited a porous structure with random orientation containing beads. The average diameter of four TPU scaffolds calculated by using SEM images is summarized in Table 3. The average diameters of 245, 360, 1063, and 798 nm were obtained for 6%, 8%, 10%, and 12% *W*/*V* TPU nanofibers. According to the previous studies [32], these results confirm that the concentration of nanofibers can significantly affect the structure of fibers, the formation of the beads, and the fiber average diameters [33]. As shown in Table 3, the average diameters of the TPU nanofibers and their uniformity were found to increase with augmented polymer concentrations (except at 12%). The formation of beads was found to increase at lower nanofiber concentrations. These variations were attributed to the changes in the viscosity of the solution. Solution viscosity is related to the augmented nanofiber chain entanglement due to the increased number of nanofiber chain molecules [34, 35].

Several investigations represented the role of nanofiber concentration in retaining the stability of the electrospun jet [36, 37]. Cross-links in a solution containing nanofiber chains are sufficiently established when the solution concentration is optimal. Otherwise, during the electrospinning process, electrospun fibers are formed with the beads [37]. The properties of the final electrospun fibers rely on solution properties such as viscoelasticity and the processing mentioned above variables. During electrospinning, a solution with low viscosity has a low viscoelastic force. It is not able to match the electrostatic and columbic repulsion forces that stretch the electrospinning fibers. This low viscosity causes the fibers to disrupt partially. Under surface tension, the high numbers of free solvent molecules in the solution come together into a spherical shape causing the formation of beads. When the solution concentration increases, an increase in viscosity occurs, causing an improvement in the viscoelastic force. Hence, partial disruption of the fibers is prevented. The augmented solution viscosity also enables the solvent molecules to be distributed over the entangled polymer molecules, leading to bead-free smooth fibers and improved fiber uniformity [38].

In this study, the average diameter of nanofibers at 12% TPU is lower than 10% TPU. It is maybe due to the lower extent of beads in higher concentrations [39]. When the concentration increased from 6% to 12%, the shape of the beads deformed from spherical to the spindle [40], the solution concentration used strongly affected the electrospinning process and the electrospinnability of nanofiber solutions. The viscosity of the solution should not be too high to move through the induced electric field. Therefore, the nanofiber concentration needs to determine adequately to avoid disruption of nanofiber structures [33].

### 3.2. FTIR Analysis of Electrospun Nanofibers

**Figure 4:**
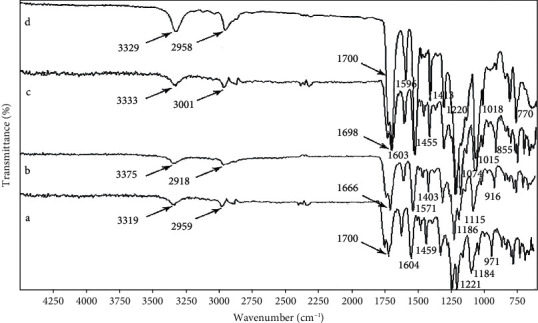
FT-IR spectra of electrospun TPU nanofibers at different concentrations: (a) 6% *W*/*V*, (b) 8% *W*/*V*, (c) 10% *W*/*V*, and (d) 12% *W*/*V*.

The FT-IR results as depicted in Figure 4 showed that absorption bands at 6% concentration of TPU are 3319, 2959, 1700, 1604, 1459, 1221, 1184, and 971 cm^−1^ which are assigned to N-H stretching (amide), C-H stretching, C=O stretching (amid and ester), C=C stretching (aromatic), CH_2_ bending, C-C stretching, C-O stretching, and long-chain bendings, respectively. A nearly similar absorption band appeared at the 8% concentration of TPU at 3375, 2918, 1666, 1571, 1403, 1186, 1115, and 916 cm^−1^. At 10% concentration of TPU, the absorption bands are 3333, 3001, 1698, 1603, 1455, 1074, 1015, and 885 cm^−1^. At 12% concentration of TPU, the absorption bands are 3329, 2958, 1700, 1596, 1413, 1220, 1018, and 770 cm^−1^. Previous studies confirmed the presence of several amid groups as a characteristic feature of polyurethan [41]. All nanofibers entail these peaks in their relevant FT-IR spectra. The representative absorption amide peaks are detected due to stretching vibration of C=O in the amide bond at around 1700 cm^−1^, bending vibration of amide bond N-H at around 1500 cm^−1^, and C–N stretching vibration amide bond at around 1220 cm^−1^ [42]. These results show that changes in concentrations do not disrupt the chemical structure of the TPU.

### 3.3. Mechanical Properties of Electrospun TPU Nanofibers

**Figure 5:**
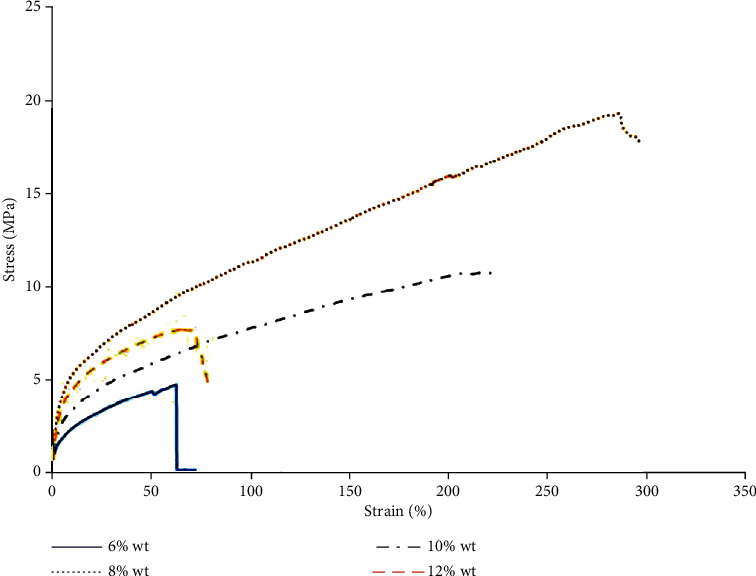
Tensile stress-strain curve of electrospun TPU nanofibers in different concentrations.

One of the most critical properties needed in tissue engineering is good mechanical properties. Mechanical stability and compatibility of the scaffolds play an essential role in the biomedical approach. Electrospun nanofibers could potentially supply useful mechanical features such as tensile strength, the breakage mechanism, and fiber morphology.  Table 4 demonstrates the mechanical properties of the electrospun scaffolds in different concentrations of TPU. The calculated values of tensile strength and elongation at break were found as 4.75 ± 0.39 MPa and 71.78 ± 7.18 mm for the 6% concentration, 19.3 ± 2.91 MPa and 295.23 ± 27.52 mm for the 8% concentration, 10.8 ± 1.94 MPa and 222.01 ± 41.77 mm for the 10% concentration, and 7.74 ± 0.21 MPa and 78.40 ± 4.46 mm for the 12% concentration, respectively. The tensile stress-strain curve of TPU nanofiber mats with various concentrations is shown in Figure 5.

The 6% TPU nanofibers were illustrated low tensile strength compared to the other nanofibers. On the other hand, 8% and 10% TPU nanofibers were found to have increased tensile strength and elongation at break value than the 12% TPU nanofibers. Therefore, it was seen that electrospinning of 8% and 10% concentrations of TPU nanofibers affected the elastic and plastic deformation under the same experimental condition. The mechanical properties results showed no significant difference in tensile strength values between the 8% and 10% concentrations of nanofibers. Lower tensile strength values in low concentrations of electrospun TPU nanofibers are due to the presence of beads in the nanofiber mats, which possess an unpleasant effect on a fiber [43]. Therefore, from the present study, it has been observed that the weak points increase with the increased beads of nanofibers. Flexible and smoother fibers with improved diameter uniformity are formed as the concentration increases [43]. This results in increased fiber cohesion points, accordingly increasing the tensile strength.

At 6% TPU, it is expected that low concentration and the presence of beads lead to lower elongation at break. Also, at 12% TPU, the higher concentration would diminish tensile properties due to the increasing diameter, causing reduced fiber surface area and decreased fiber interaction points. Both higher diameter and lower beads at 8% and 10% of TPU compared to the others account for high elongation at break. Therefore, an optimum point of average diameter and bead extent could ultimately be reached at 8% TPU. Recently, there is a report that showed a consistent result. Li et al. showed that the 8% TPU concentration in two different solvent systems resulted in smooth and uniform nanofibers [37].

### 3.4. Postsurgical Antiadhesion Potency of Electrospun TPU Nanofibers

The antiadhesion potency of TPU made from different concentrations compared to synthetic surgical mesh was evaluated by a rat cecum abrasion model. The TPU nanofibers were appropriately flexible, well-handled during surgery, and effectively protected the injured area. Untreated rats (the rat group without any postsurgical matrices) and rat groups treated with synthetic surgical mesh were considered as control and standard groups, respectively.

The tissue antiadhesion potential of the nanofibers was evaluated for 3 and 5 weeks after surgical operation macroscopically and histopathologically.

#### 3.4.1. Macroscopic Evaluation of TPU

**Figure 6:**
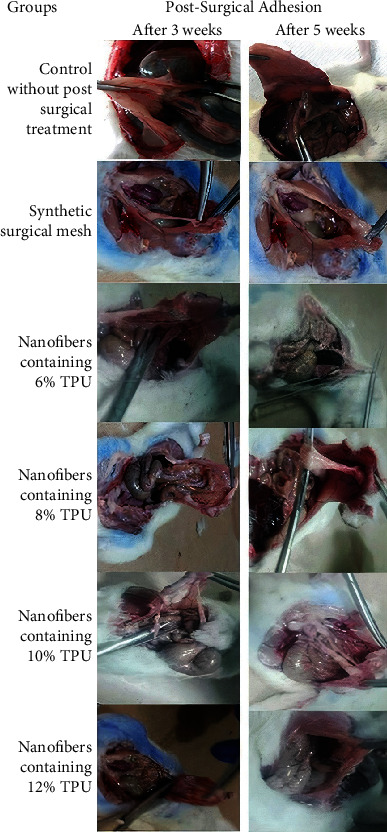
Adhesion of nanofibers in different concentrations after 3 and 5 weeks: 6%, 8%, 10%, and 12% *W*/*V* TPU compared to synthetic surgical mesh and control groups.

A digital camera was used to take photographs for macroscopic evaluation of nanofibers' effects on postsurgical adhesion in the rat's peritoneal cavity shown in Figure 6. The adhesions were graded using the adhesion scores as described in Materials and Methods. As reported in Table 2 for the macroscopic adhesion score, the control rats showed severe adhesion with large blood vessels, receiving the highest adhesion score, which was ranked 3.62 in summary after 3 weeks and 3.25 after 5 weeks using Equation (1) [28]. The adhesion score in the group treated with a synthetic surgical mesh did not decrease after 3 weeks; however, it significantly reduced after 5 weeks. Peritoneal adhesion scores significantly decreased in groups treated with nanofibers containing 6% TPU compared to control (*p* < 0.05) and received a score equivalent to 2.50 and 1.625 after 3 and 5 weeks. The scores were not significantly different compared to the synthetic surgical mesh group (*p* < 0.05). The nanofibers containing 8% and 10% TPU significantly reduced peritoneal adhesions to different extents than those of the control and synthetic surgical mesh group (*p* < 0.05) both after 3 and 5 weeks. The nanofibers containing 8% TPU remarkably inhibited adhesion formation. Adhesion scores for this group were significantly lower than those for the control group (*p* < 0.001) as well as the synthetic surgical mesh group (*p* < 0.001 and *p* < 0.01 after 3 and 5 weeks, respectively).

Interestingly, the nanofibers containing 8% and 10% TPU also inhibited inter-visceral adhesions. According to the best of our knowledge, there is no study using different TPU nanofiber concentrations to evaluate postsurgical adhesion. However, several animal studies are using other nanofibers to evaluate postsurgical adhesion. Yamaoka et al. applied a copolymer of polylactic acid- (PLGA-) polyethylene glycol (PEG) for inhibiting postsurgical adhesion [44]. Although this copolymer was rapidly inflated, concentrated, and collapsed in biological solutions, significant adhesion occurred in the cecum area since it was used as a postsurgical barrier. Macroscopic scoring of postsurgical adhesion for rats treated with PLGA nanofiber membranes loaded with epigallocatechin-3-O-gallate after a week in another study showed acceptable antiadhesion efficacy, better than untreated rats or the PLGA nanofiber-applied group. However, this nanofiber was still not a better commercial tissue adhesion barrier [45].

#### 3.4.2. Histological Evaluation of Implant

**Figure 7:**
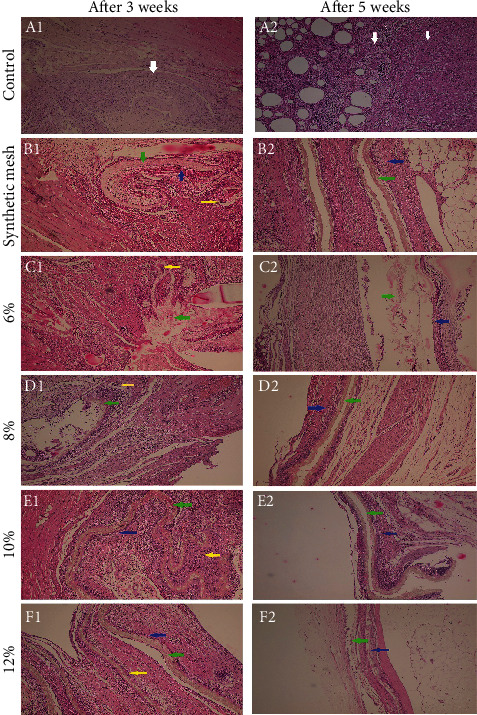
Selected pictures represent the histological observations with magnification ×100: (A1, B1, C1, D1, E1, F1) control, synthetic mesh, 6%, 8%, 10%, 12% *W*/*V* after 3 weeks; (A2, B2, C2, D2, E2, F2) control, synthetic mesh, 6%, 8%, 10%, 12% *W*/*V* after 5 weeks. White arrow: severe inflammation with fibrosis; yellow arrow: giant cell reaction; green arrow: scaffold; blue arrow: capsule. In the control group, a severe inflammatory reaction is present. The score in scaffold groups was gradually subsided over time. In a scaffold with 12% TPU, a dense capsule is formed on both sides.

Histological evaluation was performed on the specimen as previously described. A series of images of histological sections taken at magnification ×100 to represent the overall picture of the implantation site are given in Figure 7. According to the scoring system shown in Table 1, the evaluation was done in two different categories: capsule and surrounding tissue [30]. The details of the results are presented in Table 5, representing the histological scoring of animals sacrificed at two different times (after 3 and 5 weeks), and were presented as mean ± SD.

As the concentration was increased from 6% to 12%, the scores were slightly decreased. This trend was detected in specimens that were sacrificed at the 3rd and 5th weeks of implantation. As a general tendency, the scores gradually reduced with time, which means that the tissue reaction was gradually subsided. The histological pictures have represented these changes during time. Overall, regarding histopathological findings, a few points are mentioned as follows.

A severe inflammatory reaction could be seen in the control group. In a scaffold with 12% TPU, a relatively dense capsule was formed on both sides of the scaffold, gradually degraded as the macrophages and giant cells were easily seen in the membrane's pores. The capsule was always rich in capillary-like blood vessels and spindle-shape fibroblasts which surrounded the membranes. There was no difference in capsular thickness regarding the membrane's side, near the abdominal wall, or the peritoneal side. The type of infiltrated inflammatory cells was gradually changed from polymorphonuclear to lymphoblast cells, macrophages, and giant cells. Neither necrosis was observed in any of the samples at any time point.

As a result, the microscopic evaluations showed that postsurgical usage of TPU in 8, 10%, and 12% concentrations relieved abdominal adhesion, and their effects were a little better than the commercial synthetic surgical mesh. Besides, macroscopic evaluations revealed that TPU nanofibers, especially in 8% and 10% concentrations, significantly prevent abdominal adhesion compared to commercial synthetic surgical mesh. It seems that these nanofibers exerted strong fibrinolytic activity due to chemical occlusion to inhibit adhesion formation in addition to being an excellent physical barrier. Some antiadhesion agents using drug-impregnated physical barriers, paclitaxel-loaded hyaluronic acid films, and sirolimus-eluting polypropylene meshes also showed effectively prevent postsurgical adhesions [46, 47].

### 3.5. Biodegradation of Electrospun TPU Nanofibers

**Figure 8:**
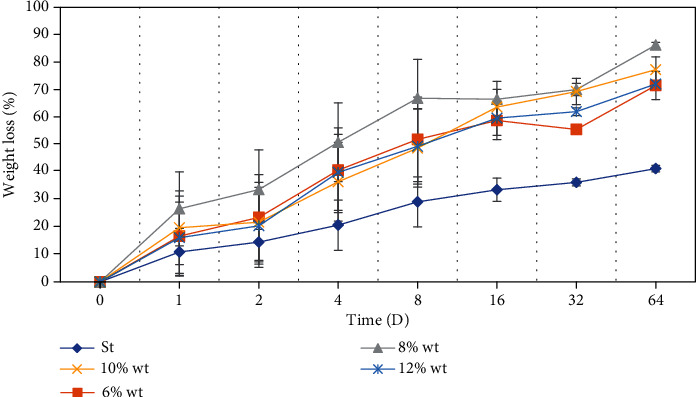
The behavior of electrospun TPU nanofibers in different concentrations (6%, 8%, 10%, 12% *W*/*V*) in weight loss experiment compared to standard surgical mesh during 64 days. The nanofibers were placed in phosphate-buffered saline and incubated at 37°C in a sealed container during the investigation.

One of the crucial issues in the design and synthesis of surgical mesh nanofibers is the time of degradation, because the degradation rate should be preferably in line with the rate of the wound healing process caused by incisions. Degradation of nanofibers usually takes several steps, including water absorption, reduction of mechanical properties (modulus and strength), removal of molar mass, and weight loss to metabolize into oligomeric components [48, 49]. A method to test the scaffold's degradation is weight loss measurement, as previously described in Materials and Methods. In this way, the in vitro degradation assessment could be consistent with the *in vivo* behavior of these scaffolds. This assay was performed over a span of 64 days (Figure 8). Obtained results indicated that electrospun TPU nanofibers represented ascending degradation kinetics all in comparison with standard surgical mesh. The tunable feature of nanofiber degradation is to match the tissue regeneration time frame at the same pace as the growth of new tissue [26]. As shown in Figure 8, weight loss reached about 50-70% after the first 8 days for all concentrations and up to 86.23% by day 64 for the 8% TPU nanofiber, which was faster than the standard surgical mesh [26–28]. 8% TPU has the higher weight loss percentage amongst other concentrations, and 6% TPU has the lower one. It is likely that the excellent wettability and increased water uptake significantly promote the degradation of 8% TPU because of its morphology.

**Figure 9:**
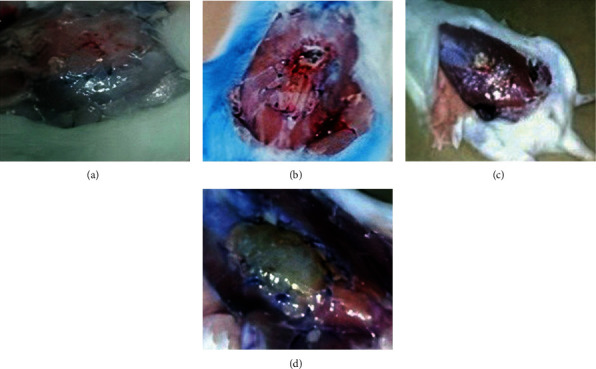
Electrospun TPU nanofiber degradation after 5 weeks postimplantation: (a) 6%, (b) 8%, (c) 10%, and (d) 12% *W*/*V*.

Our animal model created defects in the abdominal cavity after three and five weeks of postimplantation to monitor the implants.  Figure 9 represented biodegradation of electrospun TPU nanofibers which occurs at the injured area of the implanted site.

### 3.6. Antimicrobial Activity of Electrospun TPU Nanofibers

**Figure 10:**
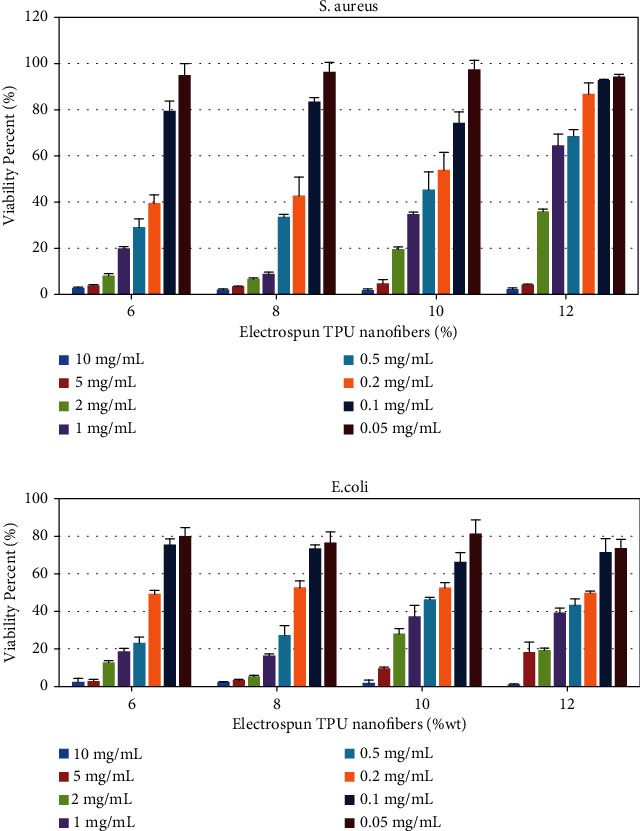
Viability percentage of *E. coli* and *S. aureus* in tested concentrations of electrospun TPU nanofibers.

Two pathogenic bacterial strains from both Gram-positive and Gram-negative groups, *S. aureus* and *E. coli*, were used as the target bacteria to screen the *in vitro* antibacterial susceptibility of the different concentrations of electrospun TPU nanofibers by the microdilution method. The compounds reveal a dose-dependent bacterial growth inhibition (Figure 10). The MIC values of the tested bacterial strains are shown in Table 6. These results represent that electrospun TPU nanofibers could have broad-spectrum antimicrobial activity in higher concentrations (10 mg/mL) for all compounds. Amongst them, 8% of electrospun TPU nanofibers exhibited more potent antibacterial activity with the lowest MIC against both bacterial strains (Table 6). Antibacterial properties of these compounds are amongst the most important properties that a substance must have to perform the best wound healing performance and play an essential role in the progression and consequences of surgery. The antibacterial effect is likely attributed to easy adherence of electrospun TPU nanofibers to the microorganisms resulting in an inhibitory effect with blocking the bacterial growth, eventually leading to their death.

## 4. Conclusions

The electrospun TPU nanofibers were fabricated in different concentrations (6%, 8%, 10%, and 12% *W*/*V*). The purpose of this study was to provide electrospun nanofibers having biodegradability and antibacterial properties that prevent any adhesions or inflammation after pelvic and abdominal surgeries. As a result, the morphology observed from SEM and AFM confirmed that the nanofibers had a uniform structure and fine morphology in higher concentrations. According to this study, the nanofiber containing 8% TPU meets the intended applications for postsurgical antiadhesive treatment. An *in vivo* study using a rat cecal abrasion model exhibited good antiadhesive properties, with biodegradation in the proper time for the nanofibers mentioned above. These electrospun TPU nanofibers had a healing effect on postsurgical adhesion compared to those of the control group, and even the standard mesh judging from macroscopic observation further confirmed the histological analysis. It seems that this valuable feature is due to the enhanced interaction between the nanofiber and body fluids, which in turn is a result of the high surface-to-area ratio of TPU nanofibers. This study also revealed that the rats did not show symptoms of fever, severe inflammatory reactions, or death in any of the experimental groups. It seems that electrospun TPU nanofibers create a physical barrier to prevent exogenous fibroblasts from entering the inflammatory area and invoking inflammatory mediators. It also helps the wound healing process by preventing the spread of the inflammatory process to nonsurgical areas [50, 51]. These nanofibers help increase the proliferation of endogenous myofibroblasts and endothelial cells by providing proper support and preventing the uncontrolled entry of the extracellular matrix [52]. These electrospun TPU nanofibers show significant antimicrobial activity. Therefore, it can be concluded that the electrospun TPU nanofibers (especially in the concentration of 8%) have an excellent antiadhesive effect which can be used as a potential abdominal barrier in surgeries with biodegradability and antibacterial properties, and it is expected to be used in clinical applications in the future. The mechanical behavior in this concentration confirmed an optimum point of average diameter and bead extent based on tensile strength and elongation at break value. Since the preparation of electrospun nanofibers for this TPU mesh was very convenient and cheap, it could improve the field of surgery.

It should be noted that these nanofibers are currently produced inexpensively and economically. However, it is essential to pay attention to the long-term clinical and inflammatory effects and unpredictable reactions, including allergic reactions, before entering the clinical arena. We will probably see more studies on the clinical applications of electrospun TPU nanofibers in the near future.

## Figures and Tables

**Table 1:** The histological scoring system consists of two categories: capsule and surrounding tissue [30].

Parameter	Score				
		4	3	2	1
Capsule localization		Capsule on two sides present	Capsule on one (lower) side present	Capsule on one (upper/dermis) side present	No capsule present
Capsule formation		Dense	Loose fibroadipose or loose adipose	Loose fibroelastic	No capsule present
Capsule cellular features	Fibroblast thickness	More than 30 layers	10–30 layers	0–10 layers	0 layer
	Fibroblast contacting surface			No	Yes
	Acute/chronic inflammatory process			Chronic	Acute
	The severity of the inflammatory process	Severe	Moderate	Mild	None
Inflammatory cell location	Inflammatory cells location	End and middle	Middle	End	None
	Macrophages contacting surface			No	Yes
	Giant cell contacting surface			No	Yes
	Polymorphonuclear leucocyte contacting surface			No	Yes
	Plasma cell contacting surface			No	Yes
	Blood vessels present			No	Yes
Capsule surrounding tissues	Acute/chronic inflammatory process			Chronic	Acute
	Severity of inflammatory process	Severe	Moderate	Mild	None
	Macrophages			No	Yes
	Giant cells			No	Yes
	Polymorphonuclear leucocytes			No	Yes
	Plasma cells			No	Yes
	Blood vessels present			No	Yes

**Table 2:** Macroscopic adhesion score results [28].

Group		Grade score					
		Score 0	Score 1	Score 2	Score 3	Score 4	SUM
1	Control 1: surgery without any postsurgical treatment after three weeks	0	0	0	1.125	2.5	3.625
2	Synthetic surgical mesh, sacrificed after three weeks postsurgery	0	0	0.25	1.5	1.5	3.25
3	Nanofibers containing 6% TPU, sacrificed after three weeks postsurgery	0	0.25	0.25	1.5	0.5	2.5^†^
4	Nanofibers containing 8% TPU, sacrificed after three weeks postsurgery	0	0.25	0.75	0.75	0	1.75^†^^∗^
5	Nanofibers containing 10% TPU, sacrificed after three weeks postsurgery	0	0.25	1	0.375	0	1.625^†^^∗^
6	Nanofibers containing 12% TPU, sacrificed after three weeks postsurgery	0	0.375	0.5	0.75	0.5	2.125
7	Control 2: Surgery without any postsurgical treatment after five weeks	0	0	0	2.25	1	3.25
8	Synthetic surgical mesh, sacrificed after five weeks postsurgery	0	0.5	0.5	0.75	0	1.75^†^
9	Nanofibers containing 6% TPU, sacrificed after five weeks postsurgery	0	0.25	1	0.375	0	1.625^†^
10	Nanofibers containing 8% TPU, sacrificed after five weeks postsurgery	0	0.375	0	0	0	0.375^†^^∗^
11	Nanofibers containing 10% TPU, sacrificed after five weeks postsurgery	0	0.375	0.5	0	0	0.875^†^
12	Nanofibers containing 12% TPU, sacrificed after five weeks postsurgery	0	0.375	0.75	0.375	0.5	2^†^

^†^Statistical difference between macroscopic grades of each group compared to control. ^∗^Statistical difference between macroscopic grades of each group compared to synthetic surgical mesh. Grade score = *n*∗*G*/*N*. *n*: number of rats with same grade score in each group; *G*: macroscopic score; *N*: total number of rats in each group.

**Table 3:** The electrospinning parameters and the TPU nanofibers characteristics at different concentrations.

Comp.	Concentration (%)	Voltage (kV)	Flow rate (mL/h)	Rotation speed (rpm)	Needle tip to collector distance (mm)	Average diameter (nm)
TPU6	6%	19	1.5	500	140	245
TPU8	8%	19	1.5	500	140	360
TPU10	10%	19	1.5	500	140	1063
TPU12	12%	19	1.5	500	140	798

**Table 4:** Mechanical tensile properties of electrospun TPU nanofibers at different concentrations.

TPU (%)	Stress (MPa)	Elongation-at-break (mm)
6%	4.75 ± 0.39	71.78 ± 7.18
8%	19.3 ± 2.91	295.23 ± 27.52
10%	10.8 ± 1.94	222.01 ± 41.77
12%	7.74 ± 0.21	78.40 ± 4.46

**Table 5:** Histological evaluation and scoring for the sacrificed postsurgery animals at different concentrations of the TPU scaffolds after 3 and 5 weeks.

	Different concentrations of the TPU scaffolds	Capsule	Surrounding capsule
After three weeks	Control group (without treatment)	20 ± 1.21	28 ± 0.91
	Synthetic mesh	18 ± 1.58	25 ± 1.41
	6% TPU nanofibers	17.75 ± 0.84	25.75 ± 2.63
	8% TPU nanofibers	14.5 ± 1.52	23.75 ± 4.57
	10% TPU nanofibers	15.5 ± 1.14	22.75 ± 2.22
	12% TPU nanofibers	14 ± 0.84	21.25 ± 2.06
After five weeks	Control group (without treatment)	15 ± 0.83	24 ± 2.35
	Synthetic mesh	12.75 ± 1.14	21.5 ± 4.20
	6% TPU nanofibers	13 ± 1.00	21 ± 3.56
	8% TPU nanofibers	12.5 ± 0.55	18 ± 1.15
	10% TPU nanofibers	10.75 ± 1.52	16.5 ± 1.29
	12% TPU nanofibers	8.5 ± 1.14	15 ± 2.71

**Table 6:** Antimicrobial activities of different concentrations of electrospun TPU nanofibers against bacteria (mg/mL).

Conc.	MIC (mg/mL)	
	S. aureus	E. coli
6%	2	5
8%	1	2
10%	5	5
12%	5	10

## Data Availability

All data used to support the findings of this study are included within the article.

## References

[1] Moris D., Chakedis J., Rahnemai-Azar A. A. (2017). Post-operative abdominal adhesions: clinical significance and advances in prevention and management. *Journal of Gastrointestinal Surgery*.

[2] Rocca A., Aprea G., Surfaro G. (2016). Prevention and treatment of peritoneal adhesions in patients affected by vascular diseases following surgery: a review of the literature. *Open Medicine*.

[3] van Steensel S., van den Hil L. C. L., Schreinemacher M. H. F., Ten Broek R. P. G., van Goor H., Bouvy N. D. (2018). Adhesion awareness in 2016: an update of the national survey of surgeons. *PLoS One*.

[4] Park H., Baek S., Kang H., Lee D. (2020). Biomaterials to prevent post-operative adhesion. *Materials*.

[5] Ergul E., Korukluoglu B. (2008). Peritoneal adhesions: facing the enemy. *International Journal of Surgery*.

[6] Stommel M. W. J., Strik C., ten Broek R. P. G., van Goor H. (2014). Efficacy and safety of the C-Qur™ film adhesion barrier for the prevention of surgical adhesions (CLIPEUS trial): study protocol for a randomized controlled trial. *Trials*.

[7] Borzouyan Dastjerdi M., Amini A., Nazari M. (2020). Novel versatile 3D bio-scaffold made of natural biocompatible hagfish exudate for tissue growth and organoid modeling. *International journal of biological macromolecules*.

[8] Gholami A., Hashemi S. A., Yousefi K. (2020). 3D nanostructures for tissue engineering, cancer therapy, and gene delivery. *Journal of Nanomaterials*.

[9] Karchin A., Simonovsky F. I., Ratner B. D., Sanders J. E. (2011). Melt electrospinning of biodegradable polyurethane scaffolds. *Acta Biomaterialia*.

[10] Mousavi S.-M., Nejad Z. M., Hashemi S. A. (2021). Bioactive agent-loaded electrospun nanofiber membranes for accelerating healing process: a review. *Membranes*.

[11] Rezaei A., Aligholi H., Zeraatpisheh Z., Gholami A., Mirzaei E. (2021). Collagen/chitosan-functionalized graphene oxide hydrogel provide a 3D matrix for neural stem/precursor cells survival, adhesion, infiltration and migration. *Journal of Bioactive and Compatible Polymers*.

[12] Ingavle G. C., Leach J. K. (2014). Advancements in electrospinning of polymeric nanofibrous scaffolds for tissue engineering. *Tissue Engineering Part B: Reviews.*.

[13] Goudarzian N., Esmaeli M., Mousavi S. (2021). Preparation physical, mechanical properties and biodegradable study of SAN/EOC/nanoclay/proteins nanocomposite. *Polymers from Renewable Resources*.

[14] Wunner F., Florczak S., Mieszczanek P., Bas O., Juan Pardo E., Hutmacher D. (2017). Electrospinning with polymer melts-state of the art and future perspectives. *Comprehensive Biomaterials II (Reference Module in Materials Science and Materials Engineering)*.

[15] Rieger K. A., Birch N. P., Schiffman J. D. (2013). Designing electrospun nanofiber mats to promote wound healing–a review. *Journal of Materials Chemistry B*.

[16] Mousavi S. M., Zarei M., Hashemi S. A. (2020). Asymmetric membranes: a potential scaffold for wound healing applications. *Symmetry*.

[17] Zarrintaj P., Moghaddam A. S., Manouchehri S. (2017). Can regenerative medicine and nanotechnology combine to heal wounds? The search for the ideal wound dressing. *Nanomedicine*.

[18] Shen Z., Lu D., Li Q., Zhang Z., Zhu Y. (2015). Synthesis and characterization of biodegradable polyurethane for hypopharyngeal tissue engineering. *Bio Med research international*.

[19] Fu Y., Wu G., Bian X., Zeng J., Weng Y. (2020). Biodegradation behavior of poly (butylene adipate-Co-terephthalate)(PBAT), poly (lactic acid)(PLA), and their blend in freshwater with sediment. *Molecules*.

[20] Farrokhi Z., Ayati A., Kanvisi M., Sillanpää M. (2019). Recent advance in antibacterial activity of nanoparticles contained polyurethane. *Journal of Applied Polymer Science*.

[21] Abbaszadegan A., Gholami A., Abbaszadegan S. (2017). The effects of different ionic liquid coatings and the length of alkyl chain on antimicrobial and cytotoxic properties of silver nanoparticles. *Iranian endodontic journal*.

[22] Gholami A., Shahin S., Mohkam M., Nezafat N., Ghasemi Y. (2015). Cloning, characterization and bioinformatics analysis of novel cytosine deaminase from *Escherichia coli* AGH09. *International Journal of Peptide Research and Therapeutics*.

[23] Gholami A., Rasoul-Amini S., Ebrahiminezhad A. (2016). Magnetic properties and antimicrobial effect of amino and lipoamino acid coated iron oxide nanoparticles. *Minerva Biotecnologica*.

[24] Ashoori Y., Mohkam M., Heidari R. (2020). Development and in vivo characterization of probiotic lysate-treated chitosan nanogel as a novel biocompatible formulation for wound healing. *BioMed Research International*.

[25] Movahedi M., Asefnejad A., Rafienia M., Khorasani M. T. (2020). Potential of novel electrospun core-shell structured polyurethane/starch (hyaluronic acid) nanofibers for skin tissue engineering: in vitro and in vivo evaluation. *International Journal of Biological Macromolecules*.

[26] Kai D., Liow S. S., Loh X. J. (2014). Biodegradable polymers for electrospinning: towards biomedical applications. *Materials Science and Engineering: C*.

[27] Azarang A., Farshad O., Ommati M. M. (2020). Protective role of probiotic supplements in hepatic steatosis: a rat model study. *BioMed Research International*.

[28] Kucukozkan T., Ersoy B., Uygur D., Gundogdu C. (2004). Prevention of adhesions by sodium chromoglycate, dexamethasone, saline and aprotinin after pelvic surgery. *ANZ journal of surgery*.

[29] Omidifar N., Nili-Ahmadabadi A., Gholami A., Dastan D., Ahmadimoghaddam D., Nili-Ahmadabadi H. (2020). Biochemical and histological evidence on the protective effects of *Allium hirtifolium Boiss* (Persian shallot) as an herbal supplement in cadmium-induced hepatotoxicity. *Evidence-Based Complementary and Alternative Medicine*.

[30] Bölgen N., Vargel İ., Korkusuz P., Menceloğlu Y. Z., Pişkin E. (2007). In vivo performance of antibiotic embedded electrospun PCL membranes for prevention of abdominal adhesions. *Journal of Biomedical Materials Research Part B: Applied Biomaterials*.

[31] Heiran R., Jarrahpour A., Riazimontazer E. (2021). sulfonamide-*β*-lactam hybrids incorporating the piperazine moiety as potential Antiinflammatory Agent with promising antibacterial activity. *ChemistrySelect*.

[32] Tarus B., Fadel N., Al-Oufy A., El-Messiry M. (2016). Effect of polymer concentration on the morphology and mechanical characteristics of electrospun cellulose acetate and poly (vinyl chloride) nanofiber mats. *Alexandria Engineering Journal*.

[33] Teo W. E., Ramakrishna S. (2006). A review on electrospinning design and nanofibre assemblies. *Nanotechnology*.

[34] Deitzel J. M., Kleinmeyer J., Harris D., Tan N. B. (2001). The effect of processing variables on the morphology of electrospun nanofibers and textiles. *Polymer*.

[35] Tungprapa S., Puangparn T., Weerasombut M. (2007). Electrospun cellulose acetate fibers: effect of solvent system on morphology and fiber diameter. *Cellulose*.

[36] Mi H.-Y., Jing X., Jacques B. R., Turng L.-S., Peng X.-F. (2013). Characterization and properties of electrospun thermoplastic polyurethane blend fibers: effect of solution rheological properties on fiber formation. *Journal of Materials Research*.

[37] Li B., Liu Y., Wei S. (2020). A solvent system involved fabricating electrospun polyurethane nanofibers for biomedical applications. *Polymers*.

[38] Mit-uppatham C., Nithitanakul M., Supaphol P. (2004). Ultrafine electrospun polyamide-6 fibers: effect of solution conditions on morphology and average fiber diameter. *Macromolecular Chemistry and Physics*.

[39] Rodoplu D., Mutlu M. (2012). Effects of electrospinning setup and process parameters on nanofiber morphology intended for the modification of quartz crystal microbalance surfaces. *Journal of Engineered Fibers and Fabrics*.

[40] Pillay V., Dott C., Choonara Y. E. (2013). A review of the effect of processing variables on the fabrication of electrospun nanofibers for drug delivery applications. *Journal of Nanomaterials*.

[41] Lopes G. H., Junges J., Fiorio R., Zeni M., Zattera A. J. (2012). Thermoplastic polyurethane synthesis using POSS as a chain modifier. *Materials Research*.

[42] Jiang L., Jiang Y., Stiadle J. (2019). Electrospun nanofibrous thermoplastic polyurethane/poly(glycerol sebacate) hybrid scaffolds for vocal fold tissue engineering applications. *Materials science & engineering C, Materials for biological applications*.

[43] Zhuo H., Hu J., Chen S., Yeung L. (2008). Preparation of polyurethane nanofibers by electrospinning. *Journal of Applied Polymer Science.*.

[44] Yamaoka T., Njatawidjaja E., Kasai A. (2013). Elastic/adhesive double-layered PLA-PEG multiblock copolymer membranes for postoperative adhesion prevention. *Polymer Degradation and Stability*.

[45] Shin Y. C., Yang W. J., Lee J. H. (2014). PLGA nanofiber membranes loaded with epigallocatechin-3-O-gallate are beneficial to prevention of postsurgical adhesions. *International Journal of Nanomedicine*.

[46] Jeong J. Y., Chung P. K., Yoo J. C. (2017). Effect of sodium hyaluronate/carboxymethyl cellulose (Guardix-sol) on retear rate and post-operative stiffness in arthroscopic rotator cuff repair patients: a prospective cohort study. *Journal of Orthopaedic Surgery*.

[47] Kimmelman C. P., Edelstein D. R., Cheng H. J. (2001). Sepragel sinus (hylan B) as a postsurgical dressing for endoscopic sinus surgery. *Otolaryngology—Head and Neck Surgery*.

[48] Hakkarainen M., Albertsson A.-C., Karlsson S. (1996). Weight losses and molecular weight changes correlated with the evolution of hydroxyacids in simulated in vivo degradation of homo-and copolymers of PLA and PGA. *Polymer Degradation and Stability*.

[49] Göpferich A. (1996). Mechanisms of polymer degradation and erosion. *Biomaterials*.

[50] Tottoli E. M., Dorati R., Genta I., Chiesa E., Pisani S., Conti B. (2020). Skin wound healing process and new emerging technologies for skin wound care and regeneration. *Pharmaceutics*.

[51] Liu H.-h., Zhang Y., Wu W., Tan X. Y. (2009). Repair of tendon injury and prevention of adhesion⋆. *Journal of Clinical Rehabilitative Tissue Engineering Research*.

[52] Anjum F., Agabalyan N. A., Sparks H. D., Rosin N. L., Kallos M. S., Biernaskie J. (2017). Biocomposite nanofiber matrices to support ECM remodeling by human dermal progenitors and enhanced wound closure. *Scientific Reports*.

